# Glutamine Administration Attenuates Poly(I:C)-Induced Lung Injury by Reducing Neutrophil Infiltration and Activating the TLR-3 Antiviral Pathway

**DOI:** 10.3390/nu17101700

**Published:** 2025-05-16

**Authors:** Li-Han Su, Wen-Chiuan Tsai, Hitoshi Shirakawa, Yu-Ling Tsai, Sung-Ling Yeh, Chiu-Li Yeh

**Affiliations:** 1 School of Nutrition and Health Sciences, College of Nutrition, Taipei Medical University, Taipei 11031, Taiwan; da07110005@tmu.edu.tw (L.-H.S.); c909228@gmail.com (Y.-L.T.); clyeh@tmu.edu.tw (C.-L.Y.); 2 Department of Pathology, Tri-Service General Hospital, Taipei 114, Taiwan; drtsaiwen@gmail.com (W.-C.T.); 3 Graduate Institute of Medical Sciences, National Defense Medical Center, Taipei 114, Taiwan; 4 Laboratory of Nutrition, Graduate School of Agricultural Science, Tohoku University, Sendai 980-8572, Japan; shirakah@tohoku.ac.jp (H.S.); 5 Department of Surgery, National Taiwan University Hospital, College of Medicine, National Taiwan University, Taipei 10002, Taiwan; sangling@tmu.edu.tw (S.-L.Y.)

**Keywords:** glutamine, alveolar–capillary barrier, TLR-3 pathway, interferon λ3, tight/adherens junction

## Abstract

**Objectives**: In this study, we investigated the effects of intravenous glutamine (GLN) administration on the Toll-like receptor 3 (TLR3) antiviral pathway and leukocyte migration in mice with poly(I:C)-induced acute lung injury (ALI). **Methods**: There were four groups in this study: the NC group, mice without an intratracheal injection; the SH group, mice intratracheally injected with endotoxin-free saline; the PS group, intratracheally instilled with 3 mg poly(I:C)/kg body weight (BW), followed by an intravenous (IV) injection of saline; and the PG group, intratracheally injected with poly(I:C) followed by the IV administration of 0.75 g GLN/kg BW. Mice in the SH, PS, and PG groups were sacrificed at 4, 12, and 24 h after intratracheal instillation. **Results**: The results showed that poly(I:C) stimulation decreased the plasma GLN concentration and increased inflammatory cytokine levels. In bronchoalveolar lavage fluid, concentrations of interferon λ3 and percentages of macrophages and M1 macrophages decreased, while neutrophils increased along with significantly elevated myeloperoxidase activity in lung tissues. The gene expressions of molecules related to leukocyte migration increased, whereas tight/adherens junction expressions in endothelial and epithelial cells were reduced. GLN supplementation upregulated the mRNA and/or protein expressions of TLR3 antiviral pathway-related factors and tight/adherens junctions while reducing inflammatory cytokines and the expressions of leukocyte migration molecules. Histological results also showed that lung injury was attenuated. **Conclusions**: These findings indicated that intravenous GLN administration after poly(I:C) instillation restored plasma GLN levels and alleviated ALI by activating the TLR3 antiviral pathway, suppressing leukocyte migration and neutrophil infiltration, mitigating inflammation, and improving the integrity of the alveolar–capillary barrier.

## 1. Introduction

Acute lung injury (ALI), classified as mild or moderate acute respiratory distress syndrome (ARDS), is a causation of morbidity and mortality in critically ill patients [1]. Viral respiratory tract infections caused by RNA viruses, including influenza viruses and coronaviruses, are common pathogens leading to ALI [2]. The excessive release of proinflammatory mediators, damage to the alveolar–capillary barrier, and the migration of accumulated immune cells to the alveoli are causes that result in ALI [3].

Macrophages and neutrophils play crucial roles in modulating inflammatory responses during lung infections [4]. Macrophages in the lungs can eliminate foreign molecules by phagocytosis and maintain pulmonary immune homeostasis by regulating inflammatory responses and tissue repair [3,5]. Neutrophils are the most abundant leukocytes in the circulation and are regarded as the first line of defense in innate immunity [6]. Prompted by chemokines released from injured pulmonary tissues, neutrophils are initially recruited to the site of inflammation to defend against pathogens [7]. Activated neutrophils may even delay the apoptotic pathway to extend their half-lives [8]. However, prolonged overactivation of neutrophils may lead to a vicious cycle by producing reactive oxygen species (ROS), granular enzymes, and proinflammatory cytokines that ultimately result in alveolar–capillary barrier injury and ALI [7]. Strategies aimed at maintaining the homeostasis of macrophages and neutrophils during lung infections are important to ameliorate ALI.

Toll-like receptors (TLRs), specifically TLR-3, play pivotal roles in recognizing viruses. TLR-3 is expressed by immune cells, fibroblasts, and epithelial cells, allowing them to recognize double-stranded (ds)RNA, an intermediate during viral replication [9]. Upon recognizing its ligands, TLR-3 initiates the Toll-interleukin (IL)-1 receptor (TIR)-domain-containing adapter-inducing interferon (IFN)-β (TRIF, also known as TICAM-1)-dependent signaling pathway. The activation of this pathway stimulates the expression of the IFN-regulatory factor 3 (IRF3) transcription factor, which results in the induction of IFN gene transcription and subsequent downstream proinflammatory chemokine production [9], which provides protection against viral infections. A previous study found that TLR-3-deficient mice had an impaired antiviral immune response during acute retroviral infections [10].

Glutamine (GLN) is the predominant free amino acid found in the plasma and tissue pool. Under normal circumstances, the body can produce GLN, which is a non-essential amino acid. However, during catabolic states such as trauma, infection, surgery, or critical illness, GLN becomes essential for supporting immunomodulatory, antioxidative, and anti-inflammatory properties. In these situations, GLN is classified as a conditionally essential amino acid [11]. In metabolic functions, GLN, as a source of nitrogen and carbon, plays a central role in nitrogen transport, facilitating the shuttle of nitrogen between different tissues. Additionally, GLN is involved in the tricarboxylic acid cycle, where it provides energy for cells, particularly immune cells, during stressful conditions. For this reason, GLN is also referred to as an immunonutrient [12]. A prior clinical trial demonstrated that GLN supplementation lowers the rate of infection, the length of hospital stays, and mortality and ameliorates inflammation in critically ill patients [13]. An animal study indicated that GLN administration impairs neutrophil migration into infection sites by reducing the concentrations of chemotactic factors [14]. Former studies carried out by our laboratory also revealed that GLN supplementation modulates the polarization of T cell subsets, alleviates local and systemic inflammation, and thus ameliorates lung injury [15,16,17,18]. However, the favorable effects of GLN in the above-mentioned studies were derived from models of lipopolysaccharide (LPS)- or polymicrobial peritonitis-induced sepsis [14,15,16,17,18]. Innate immune responses against LPS or live Gram-negative bacterial pathogens work mostly by activating the TLR4 signaling pathway [19], which is distinct from the virus-recognizing TLR-3 pathway. At present, the potential mechanism of GLN interventions against virus-induced ALI is still unclear and needs to be evaluated. Polyinosinic-polycytidylic acid (poly(I:C)) is a synthetic dsRNA known to activate the TLR-3 pathway which triggers antiviral and inflammatory responses [20]. In this study, intratracheal poly(I:C) was injected to induce ALI in mice. This animal model simulates human respiratory viral infections and enables the evaluation of nutrient interventions on molecular mechanisms in virus-induced ALI. The impacts of the administration of intravenous (IV) GLN after poly(I:C) injection on TLR-3 pathway-related antiviral responses were evaluated. Also, populations of macrophages and neutrophils, as well as inflammatory mediators in bronchoalveolar lavage fluid (BALF) were analyzed. Once a respiratory tract infection occurs, leukocyte migration to the endothelium and epithelium is regulated by adhesion molecules and chemokine receptors [21]. The overexpression of these adhesion-associated proteins promotes the interaction of leukocytes and endothelial cells and leukocytes and epithelial cells, which results in barrier damage and tissue injury [21,22]. Therefore, we isolated leukocytes, endothelial cells, and epithelial cells from lung tissues so as to explore the role of GLN administration in leukocyte migration in this model. We hypothesized that GLN administration would activate the TLR-3 antiviral pathway, decrease leukocyte migration, and alleviate intratracheal poly(I:C)-induced ALI.

## 2. Materials and Methods

### 2.1. Animals

Six-week-old male C57BL/6 mice were obtained from the National Laboratory Animal Center (NLAC, Taipei, Taiwan). All mice were raised in an environment with consistent humidity (55% ± 2%) and temperature (22 ± 2 °C), as well as a 12 h light/dark cycle. A standard rodent chow diet (cat. no. 5001, Purina Mills, St. Louis, MO, USA) was given *ad libitum* for 2 weeks before and throughout the experiment. The procedures in these experiments were approved by the Institutional Animal Care and Use Committee of Taipei Medical University (LAC2022-0227 and LAC2022-0229).

### 2.2. Experimental Procedures

After 2 weeks of adaptation, the mice were randomly allocated to four groups. The mice that received an intratracheal injection were anesthetized by an intraperitoneal (IP) injection of zoletil (25 mg/kg body weight (BW), Virbac, Carros, France) combined with rompun (10 mg/kg BW, Bayer, Leverkusen, Germany). An intratracheal injection was carried out to directly penetrate the tracheal lumen using a 30-gauge (30 G) insulin syringe (cat. no. 328838, BD, Franklin Lakes, NJ, USA). Intratracheal poly(I:C) (cat. no. tlrl-pic, InvivoGen, San Diego, CA, USA) was injected to induce ALI with 50 μL of endotoxin-free saline at a dosage of 3 mg/kg BW, followed by 100 μL of air to ensure consistent distribution into the lungs [23]. Mice intratracheally injected with 50 μL of endotoxin-free saline served as the sham controls. Afterward, half of the poly(I:C) group was administered an IV injection of 0.75 g GLN/kg BW (20% Dipeptiven, Fresenius Kabi Austria, Graz, Austria) via a tail vein immediately after the intratracheal injection, while the other half was given an equal volume of saline. The dosage of GLN used in this study was proven to be safe, has immunomodulatory effects, and was found to decrease abdominal sepsis-induced distal organ injury [15,17,18,24]. After surgery, the animals were rehydrated with 4 mL/kg of sterile saline and kept warm using a heater until the mice regained consciousness. All the mice had free access to water and chow. Pain management included administering 100 μL of 0.25% bupivacaine at the incision site before closing the skin. There were four groups in this study: (1) the normal control group (NC, n = 15): normal mice without an intratracheal injection which were sacrificed simultaneously with the sham group; (2) the sham group (SH, n = 16): mice intratracheally injected with endotoxin-free saline and sacrificed at 4 h after the injection; (3) the PS group: mice intratracheally injected with poly(I:C) and IV saline administered immediately after the poly(I:C) injection; and (4) the PG group: mice intratracheally injected with poly(I:C) and IV GLN administered immediately after the poly(I:C) injection. Mice in the PS and PG groups were sacrificed at 4, 12, and 24 h after poly(I:C) instillation (with n = 17 or 18 at each time point). All mice were sacrificed by cardiac puncture according to the experimental schedule. Upon sample collection, the mice in each group were separated into two subgroups. Half of the mice were used for collecting BALF, and the other half were used to harvest complete lung tissues without flushing. Plasma was obtained via centrifugation from blood and stored at −80 °C. The superior lobe of lung tissues was used for the histopathological analysis, and the remaining parts were stored at −80 °C for further analysis. BALF was collected via a 22G IV catheter (cat. no. SR-OX2225CA, Terumo, Tokyo, Japan) inserted into the trachea and flushed with 900 µL of ice-cold phosphate-buffered saline (PBS), followed by flushing twice with 1 mL of PBS to acquire the remaining cells in the alveoli. Supernatants were obtained from the first flush via centrifugation for 10 min at 4 °C and 1000 rpm. Cells were resuspended in PBS for flow cytometry. After flushing, lung tissues were collected and washed with ice-cold PBS in a 10 cm Petri dish for dissociation.

### 2.3. Quantification of GLN Concentration in Plasma

Ten-microliter plasma samples were used and processed using a Waters AccQ-Tag derivatization kit (cat. no. 186003836, Waters, Milford, MA, USA). The ACQUITY UPLC System (Waters) was employed for separation via ultraperformance liquid chromatography (UPLC). Monitoring was conducted using a Xevo TQ-XS mass spectrometer (Waters). GLN concentrations were measured with the Waters MassLynx 4.2 software and quantified using TargetLynx.

### 2.4. Concentrations of Inflammatory Cytokines and Chemokines in Plasma

The concentrations of inflammatory markers, including the macrophage inflammatory protein (MIP)-2, keratinocyte-derived chemokine (KC), tumor necrosis factor (TNF)-α, and interleukin (IL)-6, were determined via enzyme-linked immunosorbent assay (ELISA) kits (cat. nos. MKC00B, MM200, M6000B, and MHSTA50, R&D Systems, Minneapolis, MN, USA), with procedures performed in accordance with the supplier’s guidelines.

### 2.5. Populations of Macrophages and Neutrophils in BALF

Cells obtained from BALF were resuspended in PBS and then prepared with a LIVE/DEADTM Fixable Near-IR (infrared) Dead Cell Stain kit (cat. no. L34975, Invitrogen, Carlsbad, CA, USA) to remove non-viable cells. Viable cells were further stained for the cluster of differentiation 45.2 (CD45.2) with Pacific blue (PB, cat. no. 109820, Biolegend, San Diego, CA, USA), F4/80-brilliant violet 711 (BV711, cat. no. 123147, Biolegend), CD80-phycoerythrin (PE, cat. no. 104707, Biolegend), and Ly-6G-fluorescein (FITC, cat. no. 127605, Biolegend), which are the respective markers of leukocytes, macrophages, M1 macrophages, and neutrophils. The fluorescence minus one (FMO) control was applied for gating the cell population (Figure S1). Leukocytes, macrophages, M1 macrophages, and neutrophils were, respectively, expressed as CD45.2^+^, CD45.2^+^/F4/80^+^, CD45.2^+^/F4/80^+^/CD80^+^, and CD45.2^+^/Ly-6G^+^ cells.

### 2.6. Isolating Leukocytes, Endothelial Cells, and Epithelial Cells from Lung Tissues

After collecting BALF, lung tissues were dissociated via a mouse lung dissociation kit (no. 130-095-927, Miltenyi Biotec, Bergisch Gladbach, Germany) and a gentleMACS Dissociator (no. 130-096-427, Miltenyi Biotec). Procedures followed instructions provided by the manufacturer. First, an enzyme mix (2.4 mL of 1× buffer S, 100 µL of enzyme D, and 15 µL of enzyme A) was prepared in a gentleMACS C tube (no. 130-093-237, Miltenyi Biotec). A lung was cut into single lobes in a Petri dish containing PBS. Then, single lobes were transferred to a C tube containing 2.515 mL of the enzyme mix; the gentleMACS Dissociator was applied, and the 37C_m_LDK_1 Program was run. At the end of the program, the sample was spun down, applied to a MACS SmartStrainer (70 µm, 130-098-462, Miltenyi Biotec), and washed with 2.5 mL of 1× buffer S. The cell suspension was centrifuged for 10 min at 300× *g*. Cell pellets were collected, resuspended in PBS, and stained with the LIVE/DEADTM Fixable Near-IR Dead Cell Stain kit (cat. no. L34975, Invitrogen) to eliminate dead cells. After washing with the cell staining buffer (no. 420201, Biolegend), cells were stained with the CD45.2, CD31, and CD326 antibodies, which are the respective markers of leukocytes, endothelial cells, and epithelial cells. After incubation and washing, cells were sorted using BD FACS AriaIII (BD Biosciences, San Jose, CA, USA). Leukocytes, endothelial cells, and epithelial cells were, respectively, identified as CD45.2^+^, CD45.2^−^/CD31^+^, and CD45.2^−^/CD326^+^ cells.

### 2.7. Concentrations of Inflammatory Cytokines and Chemokines in BALF

KC, MIP-2, IL-6, TNF-α, interferon (IFN)-β (respective cat. nos. MKC00B, MM200, M6000B, MTA00B, and MIFNB0, R&D Systems), and IFN-λ3 (cat. no. 88-7284-22, Invitrogen) were analyzed using ELISA kits. These analyses were performed according to instructions provided by the manufacturer. The Bradford protein assay reagent (cat. no. 5000006, Bio-Rad, Hercules, CA, USA) was used for protein quantification. All results were corrected according to protein concentrations and expressed as pg/mg protein.

### 2.8. Protein Expression of Phosphorylated (p)-IRF3 in Lung Tissues

Lung tissues (30 mg) were lysed in the Tissue Protein Extraction Reagent (cat. no. 78510, T-PER™, ThermoFisher Scientific, Waltham, MA, USA) with a protease and phosphatase inhibitor (cat. no. 78442, ThermoFisher Scientific) to extract total protein. Following centrifugation for 10 min at 4 °C and 12,000 rpm, supernatants were collected for further analysis. Protein concentrations were quantified as described above. In total, 200 μg of protein was subjected to an analysis of *p*-IRF3 using a PathScan^®^ RP Phospho-IRF-3 (Ser379) Sandwich ELISA Kit (cat. no. 54395, Cell Signaling, Danvers, MA, USA). Analyses were conducted following the supplier’s guidance. Results are expressed as optical density (OD) values measured at 450 nm.

### 2.9. The Preparation of Messenger (m)RNA and Analysis of a Real-Time Reverse-Transcription (RT) Quantitative Polymerase Chain Reaction (qPCR)

The total RNAs of leukocytes, endothelial cells, and epithelial cells isolated from lung tissues were extracted via an RNeasy Mini Kit (cat. no. 74106, Qiagen, Hilden, Germany). A RevertAid™ first-strand complementary (c)DNA synthesis kit (cat. no. K1622, ThermoFisher Scientific) was applied for cDNA synthesis from total RNA according to guidance provided by the manufacturer. mRNA expressions were amplified via an RT-PCR through a QuantStudio™ 1 Real-Time PCR System (Applied Biosystems, Foster City, CA, USA) with the SYBR^®^ Green Reagent (ThermoFisher Scientific). The genes involved in this study include factors related to the TLR-3 pathway, such as *IFN-stimulated gene 15* (*ISG15*) and *ubiquitin-specific peptidase 18* (*USP18*). Additionally, leukocyte migration-associated factors were examined, including *β2-integrin*, *C-X-C motif chemokine receptor 2* (*CXCR2*), *intercellular adhesion molecule 1* (*ICAM-1*), *galectin-9*, and *CD47*. The study also focused on factors related to endothelial and epithelial integrity, specifically *claudin-5*, *claudin-18.1*, and *vascular endothelial* (*VE)-cadherin*. Primers used in this study are presented in Table S1 and were obtained from Mission Biotech (Taipei, Taiwan) based on deposited cDNA sequences (GenBank database, NCBI). According to the supplier’s guidance, the Maxima SYBR Green/ROX qPCR Master Mix (2×) (ThermoFisher Scientific) with 200 ng of cDNA and 200 nM of each primer was used for amplification. Subsequently, a final dissociation curve (DC) analysis was performed. Relative mRNA expressions were analyzed using cycle threshold (CT, 2^−ΔΔCt^) values and normalized to mouse GAPDH.

### 2.10. Analysis of Myeloperoxidase (MPO) Activities in Lung Tissues

Lung tissues (10 mg) were used and lysed in the buffer from the Myeloperoxidase Activity Assay Kit (cat. no. Ab105136, Abcam, Cambridge, MA, USA). Supernatants were collected for an MPO activity analysis according to the protocol provided by the manufacturer. Protein concentrations were determined using the Bradford protein assay reagent (cat. no. 5000006, Bio-Rad). Data are expressed as mU/mg of protein.

### 2.11. Hematoxylin and Eosin (H&E) Staining and Histopathological Scoring of Lung Tissues

The superior lobe of lung tissues was fixed in 10% neutral-buffered formalin. After processing through serial dehydration, specimens were paraffin-embedded and sliced into 3 μm thick sections. Slides were stained with H&E and viewed with optical microscopy. Images were captured at 400× magnification. The lung injury score was determined based on four parameters including immune cell infiltration, alveolar or interstitial edema, hemorrhaging, and septal thickening. We used single-blind slide reading to calculate the injury score. Each parameter was scored 0–3 points (0 represents no lesion, 1 represents 1–10% of the area affected, 2 represents 11–25% of the area affected, and 3 represents 25–45% of the area affected). The total score ranged from 0 to 12.

### 2.12. Statistical Analysis

Data are expressed as the mean ± standard error of the mean (SEM). Statistical analyses were performed using the GraphPad Prism 5 software (GraphPad Software, La Jolla, CA, USA). Student’s *t*-test was used to analyze differences between the NC and SH groups. A two-way analysis of variance (ANOVA) followed by a Bonferroni *post hoc* test was used to analyze differences among the SH, PS, and PG groups at each time point. A *p* value of <0.05 was considered statistically significant.

## 3. Results

### 3.1. BWs and Weights of Lung Tissues/BW Ratio

There were no significant differences in BWs among all groups during the experimental period. In the PS group, the lung tissue weight adjusted for body weight (lung/BW ratio) was significantly higher than that in the SH group at both 12 and 24 h. However, no statistically significant differences were observed between the PS and PG groups at any time point (Table S2).

### 3.2. Concentration of GLN in Plasma

**Figure 1:**
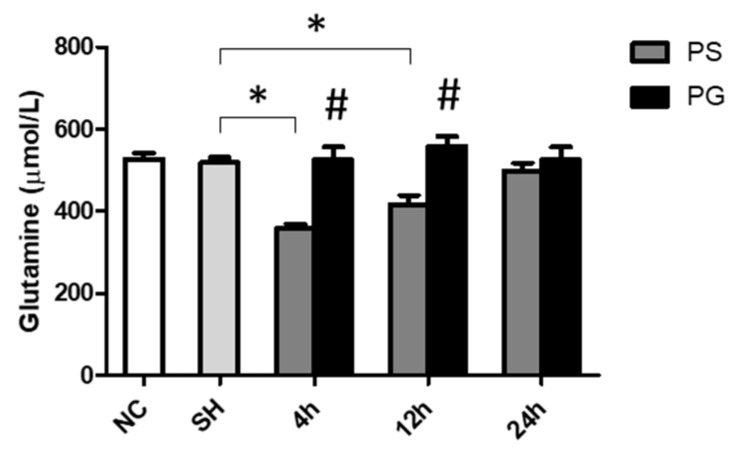
Glutamine concentrations in plasma. NC group, the normal control group; SH group, intratracheally administered saline; PS group, administered poly(I:C) intratracheally and an IV injection of saline; PG group, administered poly(I:C) intratracheally and an IV injection of glutamine. Data are expressed as the mean ± standard error of the mean (SEM). The differences between the NC and SH groups were analyzed using Student’s *t*-test. A two-way analysis of variance (ANOVA) followed by a Bonferroni *post-hoc* test was used to analyze the differences among the SH and PS groups at three time points and between the PS and PG groups at identical time points. * Significantly different from the SH group (*p* < 0.05). ^#^ Significantly different from the PS group at the identical time point (*p* < 0.05).

There were no differences in plasma GLN levels between the NC and SH groups. However, the PS group had lower GLN levels than the SH group at 4 and 12 h. Additionally, PG showed significantly higher GLN levels than the PS group at both time points (Figure 1).

### 3.3. Concentrations of KC, MIP-2, IL-6, and TNF-α in Plasma

**Figure 2:**
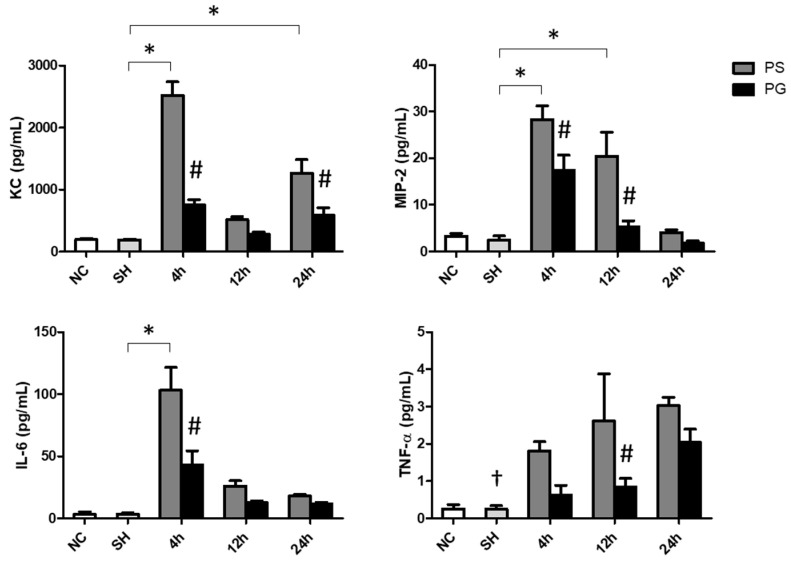
Concentrations of inflammatory chemokines and a cytokine in plasma. NC group, the normal control group; SH group, intratracheally administered saline; PS group, administered poly(I:C) intratracheally and an IV injection of saline; PG group, administered poly(I:C) intratracheally and an IV injection of glutamine. IL-6, interleukin-6; KC, keratinocyte-derived chemokine; MIP-2, macrophage inflammatory protein 2; TNF-α, tumor necrosis factor-α. Data are expressed as the mean ± standard error of the mean (SEM). The differences between the NC and SH groups were analyzed using Student’s *t*-test. A two-way analysis of variance (ANOVA) followed by a Bonferroni *post-hoc* test was used to analyze the differences among the SH and PS groups at three time points and between the PS and PG groups at identical time points. ^†^ Significantly different from the PS group at three time points (*p* < 0.05). * Significantly different from the SH group (*p* < 0.05). ^#^ Significantly different from the PS group at the identical time point (*p* < 0.05).

The sham operation did not increase the levels of inflammatory cytokines in the plasma. After poly(I:C) stimulation, the concentrations of KC were significantly increased at 4 and 24 h, IL-6 at 4 h, MIP-2 at 4 and 12 h, and TNF-α at all time points. GLN administration significantly decreased the levels of these parameters (Figure 2).

### 3.4. Concentrations of Chemokines and Cytokines in BALF

**Figure 3:**
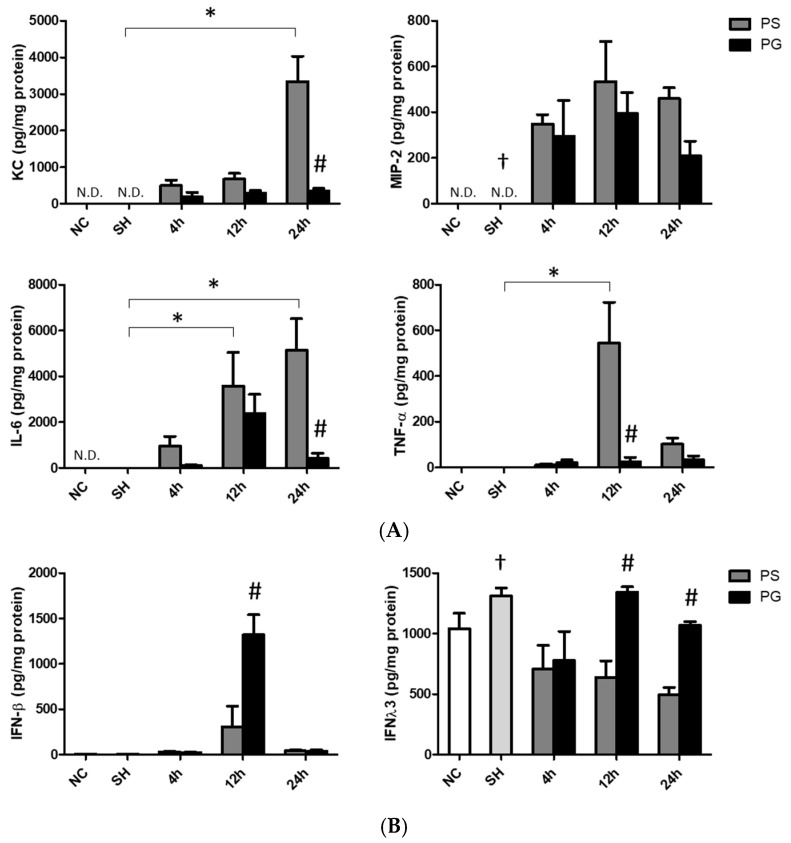
Concentrations of (**A**) inflammatory chemokines and cytokines and (**B**) antiviral cytokines in bronchoalveolar lavage fluid (BALF). NC group, the normal control group; SH group, intratracheally administered saline; PS group, administered poly(I:C) intratracheally and an IV injection of saline; PG group, administered poly(I:C) intratracheally and an IV injection of glutamine. IL-6, interleukin-6; IFN-β, interferon-β; IFN-λ3, interferon-λ3; KC, keratinocyte-derived chemokine; MIP-2, macrophage inflammatory protein 2; N.D., not detected; TNF-α, tumor necrosis factor-α. Data are expressed as the mean ± standard error of the mean (SEM). The differences between the NC and SH groups were analyzed using Student’s *t*-test. A two-way analysis of variance (ANOVA) followed by a Bonferroni *post-hoc* test was used to analyze the differences among the SH and PS groups at three time points and between the PS and PG groups at identical time points. ^†^ Significantly different from the PS group at three time points (*p* < 0.05). * Significantly different from the SH group (*p* < 0.05). ^#^ Significantly different from the PS group at the identical time point (*p* < 0.05).

The sham operation did not affect chemokines and cytokines levels in the BALF. Poly(I:C) stimulation significantly increased the concentrations of KC at 24 h, MIP-2 at all three time points, IL-6 at 12 and 24 h, and TNF-α at 12 h compared to those of the SH group. The PG group had significantly lower KC and IL-6 levels at 24 h and TNF-α at 12 h than those in the PS groups after poly(I:C) stimulation (Figure 3A). Compared to the SH group, IFN-λ3 levels were lower at all time points after poly(I:C) stimulation. For groups treated with poly(I:C), the PG group had higher levels of IFN-β at 12 h and IFN-λ3 at 12 and 24 h than the PS groups (Figure 3B).

### 3.5. Distributions of Leukocytes, Macrophages, M1 Macrophages, and Neutrophils in BALF

**Figure 4:**
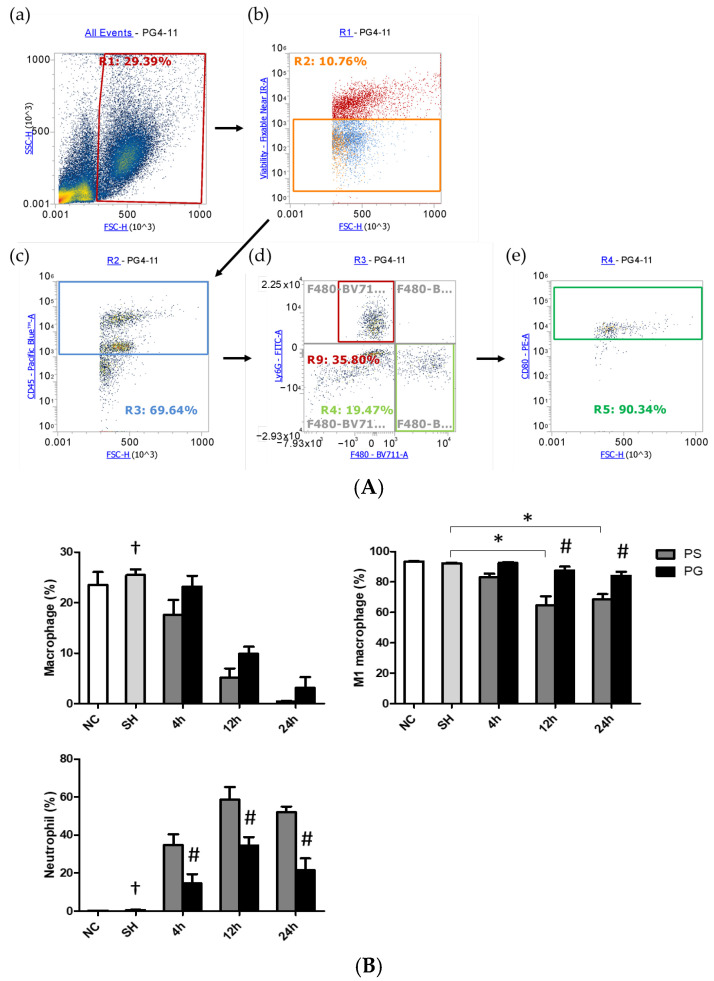
(**A**) Representative illustration of the strategy for gating leukocytes, macrophages, and neutrophils using flow cytometry. The cells were first gated to eliminate the debris (a). The live cells were chosen via cell viability dye (b). Then the antibody CD45 was used to determine leukocytes in the live cells (c). Ly6G and F4/80 were applied to gate neutrophils and macrophages, respectively, in the leukocytes (d). Among macrophages, the antibody CD80 was utilized to determine M1 macrophages (e). (**B**) Percentages of macrophages, M1 macrophages, and neutrophils in bronchoalveolar lavage fluid (BALF). Leukocytes, macrophages, M1 macrophages, and neutrophils were, respectively, identified as CD45.2^+^, CD45.2^+^/F4/80^+^, CD45.2^+^/F4/80^+^/CD80^+^, and CD45.2^+^/Ly6−G^+^ cells. NC group, the normal control group; SH group, intratracheally administered saline; PS group, administered poly(I:C) intratracheally and an IV injection of saline; PG group, administered poly(I:C) intratracheally and an IV injection of glutamine. Data are expressed as the mean ± standard error of the mean (SEM). The differences between the NC and SH groups were analyzed using Student’s *t*-test. A two-way analysis of variance (ANOVA) followed by a Bonferroni *post-hoc* test was used to analyze the differences among the SH and PS groups at three time points and between the PS and PG groups at identical time points. ^†^ Significantly different from the PS group at three time points (*p* < 0.05). * Significantly different from the SH group (*p* < 0.05). ^#^ Significantly different from the PS group at the identical time point (*p* < 0.05).

An illustration of the strategy for gating leukocytes, macrophages, and neutrophils in flow cytometry is shown in Figure 4A. The R1 region indicated that the cells were gated to eliminate debris. Next, live cells were selected using a cell viability dye from the R1 region, as shown in the orange square in panel (b). In panel (c), the antibody CD45 was utilized to identify leukocytes among the live cells, with the blue square highlighting CD45^+^ cells. Subsequently, Ly6G and F4/80 antibodies were applied to gate neutrophils (CD45^+^/Ly6G^+^) and macrophages (CD45^+^/F4/80^+^) in panel (d), respectively. Finally, panel (e) displays the CD45.2^+^/F4/80^+^/CD80^+^ cells that were selected from the CD45^+^/F4/80^+^ population identified in panel (d). The percentages of macrophages were significantly lower at all time points in the PS group compared to the SH group; however, there were no significant differences between the PS and PG groups at each time point. The population of M1 macrophages in the SH group was significantly higher than that in the PS group at 12 and 24 h. Nevertheless, at 12 and 24 h, the percentage of M1 macrophages in the PS group was lower than that in the PG group after poly(I:C) stimulation. In contrast, the percentages of neutrophils were significantly elevated following poly(I:C) stimulation. Compared to the PS group, the percentages of neutrophils in the PG group decreased after poly(I:C) stimulation (Figure 4B).

### 3.6. mRNA Expressions of Leukocyte Migration-Related Factors in Lung Leukocytes, Endothelial Cells, and Epithelial Cells

**Figure 5:**
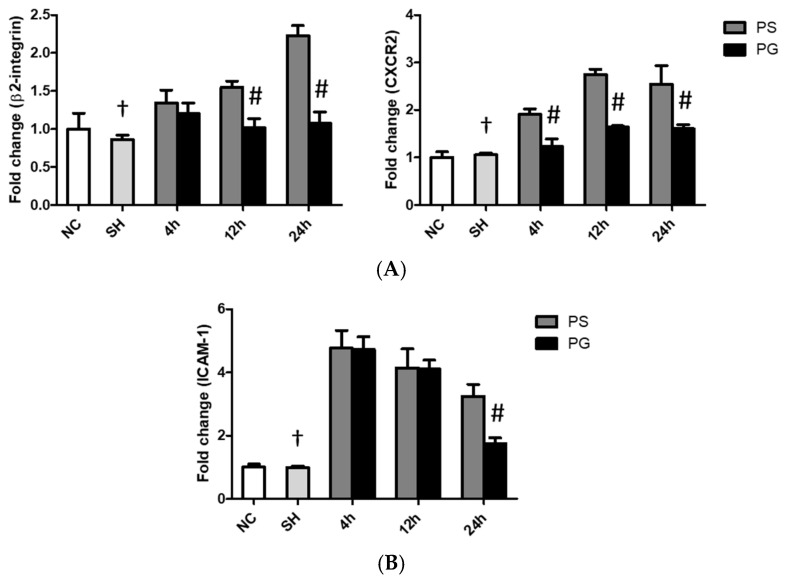

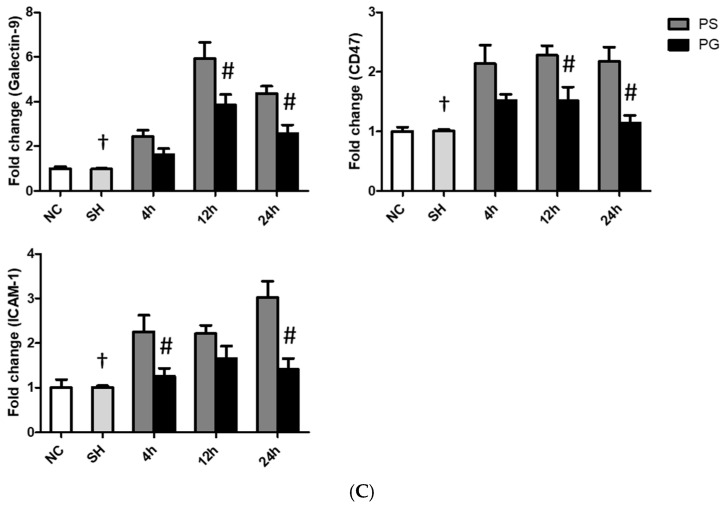
Messenger (m)RNA expressions of leukocyte migration-related factors in (**A**) leukocytes, (**B**) endothelial cells, and (**C**) epithelial cells of the lungs. NC group, the normal control group; SH group, intratracheally administered saline; PS group, administered poly(I:C) intratracheally and an IV injection of saline; PG group, administered poly(I:C) intratracheally and an IV injection of glutamine. CD47, cluster of differentiation 47; CXCR2, C-X-C motif chemokine receptor 2; ICAM-1, intercellular adhesion molecule-1. Data are expressed as the mean ± standard error of the mean (SEM). The differences between the NC and SH groups were analyzed using Student’s *t*-test. A two-way analysis of variance (ANOVA) followed by a Bonferroni *post-hoc* test was used to analyze the differences among the SH and PS groups at three time points and between the PS and PG groups at identical time points. ^†^ Significantly different from the PS group at three time points (*p* < 0.05). ^#^ Significantly different from the PS group at the identical time point (*p* < 0.05).

The sham operation had no impact on the mRNA expression of migration indicators compared to the PS group across all time points. mRNA expressions of *β2-integrin* and *CXCR2* in leukocytes, *ICAM-1* in endothelial cells, and *galectin-9*, *CD47*, and *ICAM-1* in epithelial cells in the PS group were significantly higher than those in the SH group at all three time points. Compared to the PS group, all these factors were significantly downregulated in the PG group at some or all three of the time points after poly(I:C) stimulation (Figure 5).

### 3.7. mRNA Expressions of TLR-3 Pathway-Related Factors and Protein Expressions of p-IRF3 in Lung Tissues

**Figure 6:**
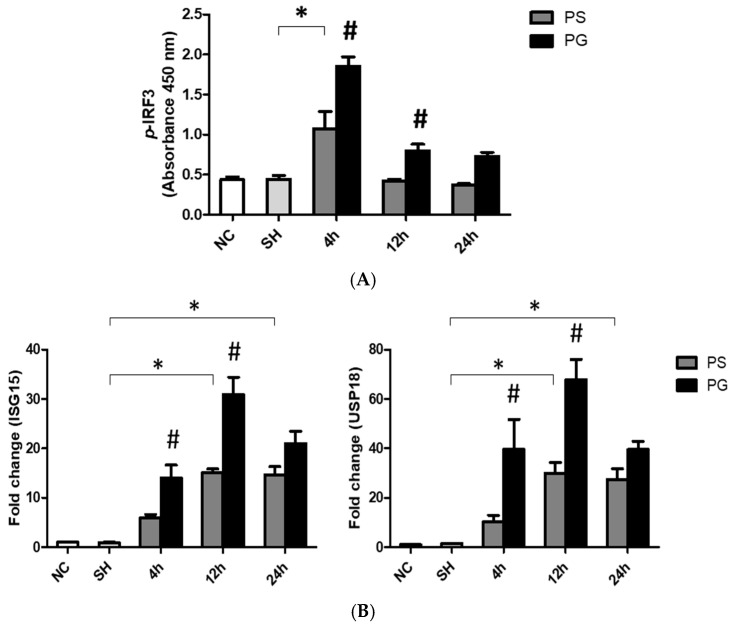
(**A**) Protein expressions of phosphorylated IFN-regulatory factor 3 (*p*-IRF3) and messenger (m)RNA expressions of (**B**) Toll-like receptor 3 (TLR-3) pathway-related factors ISG15 and USP18 in lung tissues. NC group, the normal control group; SH group, intratracheally administered saline; PS group, administered poly(I:C) intratracheally and an IV injection of saline; PG group, administered poly(I:C) intratracheally and an IV injection of glutamine. ISG15, interferon-stimulated gene 15; TRIF, Toll-IL-1 receptor (TIR)-domain-containing adapter-inducing IFN-β; USP18, ubiquitin-specific peptidase 18. Data are expressed as the mean ± standard error of the mean (SEM). The differences between the NC and SH groups were analyzed using Student’s *t*-test. A two-way analysis of variance (ANOVA) followed by a Bonferroni *post-hoc* test was used to analyze the differences among the SH and PS groups at three time points and between the PS and PG groups at identical time points. * Significantly different from the SH group (*p* < 0.05). ^#^ Significantly different from the PS group at the identical time point (*p* < 0.05).

After stimulation with poly(I:C), the protein levels of *p*-IRF3 were significantly higher in the PS group compared to the SH group at 4 h. Compared to the PS group, the PG group at both 4 and 12 h showed a significant increase (Figure 6A). Additionally, the mRNA expression of *ISG15* in the PS group was significantly upregulated compared to the SH group at both 12 and 24 h. The mRNA expression of *USP18* also increased at 12 and 24 h post-poly(I:C) induction compared to that in the SH group. In contrast, *ISG15* and *USP18* were significantly upregulated in the PG group at 4 and 12 h when compared to the PS group (Figure 6B).

### 3.8. mRNA Expressions of Adherens and Tight Junction Proteins in Lung Endothelial and Epithelial Cells

**Figure 7:**
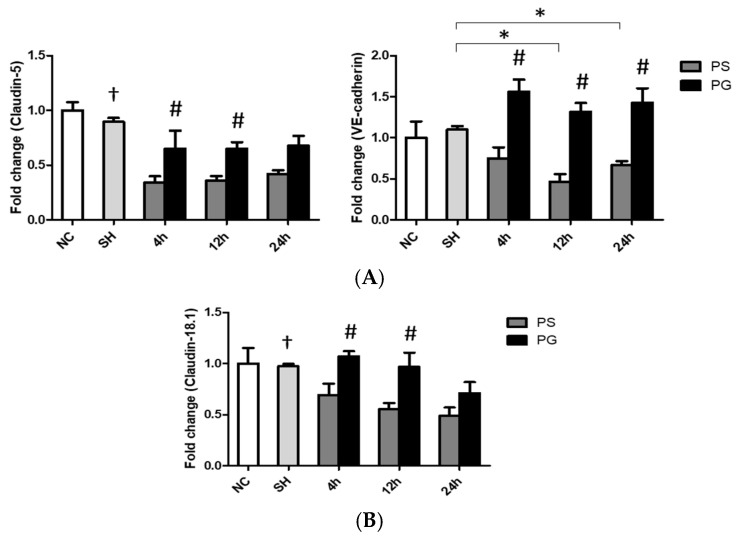
Messenger (m)RNA expressions of tight/adherens junction proteins in (**A**) endothelial and (**B**) epithelial cells of lung tissues. NC group, the normal control group; SH group, intratracheally administered saline; PS group, administered poly(I:C) intratracheally and an IV injection of saline; PG group, administered poly(I:C) intratracheally and an IV injection of glutamine. VE-cadherin, vascular endothelial cadherin. Data are expressed as the mean ± standard error of the mean (SEM). The differences between the NC and SH groups were analyzed using Student’s *t*-test. A two-way analysis of variance (ANOVA) followed by a Bonferroni *post-hoc* test was used to analyze the differences among the SH and PS groups at three time points and between the PS and PG groups at identical time points. ^†^ Significantly different from the PS group at three time points (*p* < 0.05). * Significantly different from the SH group (*p* < 0.05). ^#^ Significantly different from the PS group at the identical time point (*p* < 0.05).

In endothelial cells, the mRNA expression of *claudin-5* at all time points, as well as *VE-cadherin* at 12 h and 24 h, was higher in the SH group compared to that in the PS group. GLN administration reversed the downregulated expressions of *claudin-5* and *VE-cadherin* after poly(I:C) stimulation (Figure 7A). As for epithelial cells, poly(I:C) stimulation significantly reduced the mRNA expression of *claudin18.1*, whereas GLN administration significantly reversed the expression of *claudin18.1* to levels comparable to those in the SH group (Figure 7B).

### 3.9. Histopathological Findings of Lung Tissues

**Figure 8:**
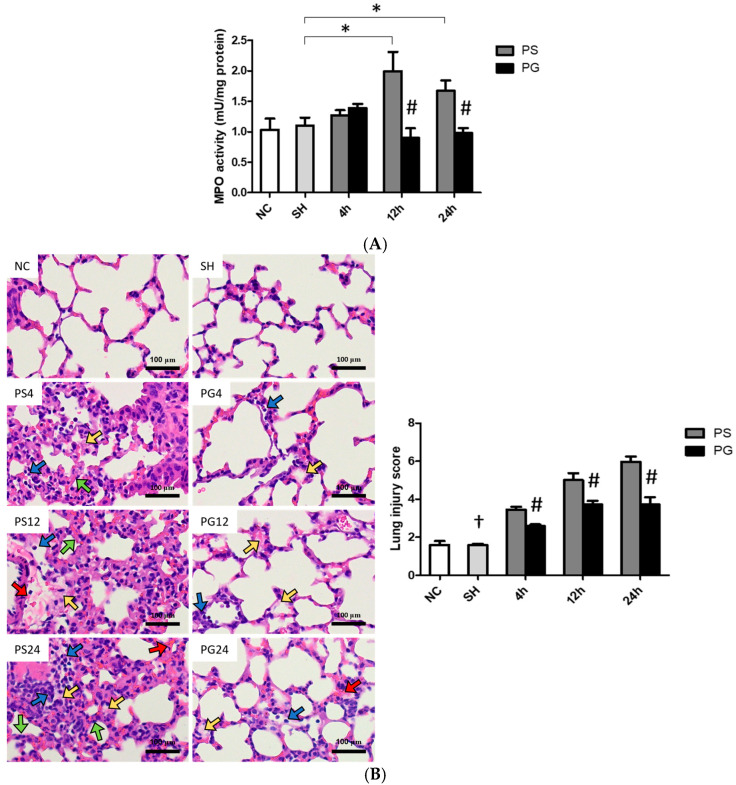
(**A**) Myeloperoxidase (MPO) activities, (**B**) representative images of hematoxylin and eosin (H&E) staining (magnification, ×400), and histopathology scores of lung tissues. Blue, yellow, red, and green arrows represent immune cell infiltration, alveolar or interstitial edema, hemorrhage, and septal thickening, respectively. NC group, the normal control group; SH group, intratracheally administered saline; PS group, administered poly(I:C) intratracheally and an IV injection of saline; PG group, administered poly(I:C) intratracheally and an IV injection of glutamine. Data are expressed as the mean ± standard error of the mean (SEM). The differences between the NC and SH groups were analyzed using Student’s *t*-test. A two-way analysis of variance (ANOVA) followed by a Bonferroni *post-hoc* test was used to analyze the differences among the SH and PS groups at three time points and between the PS and PG groups at identical time points. ^†^ Significantly different from the PS group at three time points (*p* < 0.05). * Significantly different from the SH group (*p* < 0.05). ^#^ Significantly different from the PS group at the same time point (*p* < 0.05).

MPO activities in the PS group were significantly higher than those in the SH group, while the PG group had significantly lower MPO activities than the PS group at 12 and 24 h after poly(I:C) stimulation (Figure 8A). Compared to the SH group, poly(I:C) stimulation increased immune cell infiltration, edema in the alveolar or interstitial space, hemorrhaging, and septal thickening. Poly(I:C) instillation significantly increased lung injury scores, while GLN treatment significantly reduced lung injuries (Figure 8B).

## 4. Discussion

Virus-induced pneumonia and ARDS are highly related to mortality in critically ill patients [25,26]. In this study, poly(I:C) intratracheal instillation was used to mimic ALI triggered by viral infection. The findings indicated that GLN administration alleviated poly(I:C)-induced inflammation and enhanced barrier junction expression, and histological findings suggest that lung injury was attenuated. The activation of the TLR-3-antiviral pathway and a reduction in neutrophil immigration may play regulatory roles in mitigating poly(I:C)-induced ALI.

In this study, we analyzed the populations of neutrophils and macrophages in BALF. In the early stage of respiratory infection, alveolar macrophages are activated to produce proinflammatory mediators and recruit neutrophils to the site of inflammation. However, overactivated macrophages may lead to apoptosis and programmed necrotic cell death that result in immune dysfunction and organ injury [27,28]. A previous study also reported that there was a negative correlation between the viral load and macrophage activation, and the depletion of resting macrophages was found in response to a respiratory viral infection [29]. Meanwhile, the responses of M1 macrophages were also suppressed. M1 macrophages play a critical role in reducing viral replication by producing ROS and cytokines during viral infections [30,31]. Our findings did show decreased percentages of total and M1 macrophages as well as elevated neutrophils after poly(I:C) stimulation. In this study, leukocyte migration-associated factors were measured. Leukocyte chemotaxis into the lungs plays a key role in the pathogenesis of ALI. During respiratory infections, chemokines are secreted from alveolar macrophages and other cells in the alveolar environment, which in turn results in leukocyte migration into the pulmonary interstitium. There are intercellular interactions between leukocytes and the capillary endothelium, as well as leukocytes with the alveolar epithelium. The interrelationship of inflammatory cytokines, adhesion molecules, chemokines, and their receptors orchestrate the recruitment of leukocytes into the lungs [32]. Human IL-8, along with its murine functional equivalents, KC and MIP-2, is a potent inducer of leukocyte migration into inflamed tissues [33,34]. CXCR2 on the leukocyte surface is activated by KC and MIP-2 and promotes the adherence of β2-integrin to ICAM-1 expressed on the surface of endothelial cells, which facilitates leukocyte extravasation into the interstitial space [35,36]. The transepithelial passage of neutrophils from the interstitium into alveoli depends on several molecules that are involved with adhesion, migration, and post-migration detachment. Galactin-9 is an epithelial-derived factor that attracts neutrophils to attach to epithelial cells across the epithelium via the regulation of CD47 and ICAM-1 [37,38]. CD47, a transmembrane protein expressed by neutrophils and epithelial cells, participates in neutrophil trafficking to the lungs [37]. ICAM-1 on the surface of alveolar epithelial cells is one of the ligands for integrins that are involved in leukocyte post-migration retention/detachment [37]. The findings of this study revealed that GLN administration downregulated the poly(I:C)-induced expression of chemotactic molecules expressed by leukocytes, the endothelium, and the epithelium, suggesting that leukocyte migration decreased. Neutrophil infiltration into the alveoli is a key characteristic of ALI, significantly contributing to disease progression. Upon activation, neutrophils release MPO, an enzyme that catalyzes the production of hypochlorous acid (HOCl), a potent oxidant. HOCl induces oxidative stress and tissue damage, exacerbating pulmonary inflammation and worsening clinical outcomes. MPO serves as a reliable biomarker for evaluating the extent of lung tissue injury [39,40]. Our findings indicate that GLN administration following poly(I:C) stimulation resulted in reduced MPO activity and lower neutrophil infiltration within lung tissues, suggesting a protective role against excessive immune responses. These results indicate the potential of GLN as a therapeutic intervention for mitigating neutrophil-mediated lung injury in ALI.

Viral infection activates the TLR-3 signaling pathway and the phosphorylation of IRF-3, which leads to its translocation to the nucleus. *p*-IRF3 induces type I and type III *IFN* gene transcription [9]. Both type I and type III IFNs enhance the production of numerous interferon-stimulated genes (ISGs) [41]. ISGs can induce antiviral responses by inhibiting viral replication and spread, stimulating immune cells, and promoting infected cell death [42]. IFN-β is a subtype of type I IFN. Type I IFN signaling can be triggered by the binding of IFN-β to the IFN-α/β receptor which subsequently modulates ISG transcription. *ISG15* is one of the most commonly induced genes by type I IFN signaling, and it plays a critical role in defending against viral infections [43]. USP18 is an ISG15-specific protease which negatively regulates type I IFN signaling [44]. A previous study reported that intracellular ISG15 is not only responsible for antiviral immunity but also prevents USP-dependent IFN-α/β over-amplification and auto-inflammation [45]. Compared to type I IFNs, type III IFNs exhibit a longer-lasting antiviral state and fewer proinflammatory responses [42]. An animal study demonstrated that type I IFNs are essential for controlling the spread of systemic viral infections, while type III IFNs are sufficient to protect against respiratory viral infections confined to the lung epithelium [46]. IFN-λ3 is one of the type III IFNs that promotes the expression of ISGs and contributes critically to the maintenance of respiratory mucosal integrity. IFN-λ3 offers localized immune protection, primarily focusing on epithelial cells to strengthen the barrier function and prevent viral entry, thereby minimizing systemic inflammation [47,48]. In this study, GLN administration after poly(I:C) stimulation resulted in significantly higher *p*-IRF3 levels, elevated IFN-β and IFN-λ3 concentrations in BALF, and the upregulation of type I IFN signaling pathway-related ISG15 and USP18 at the early stage after stimulation. Since ISG15 expression can inhibit the over-inflammation exerted by USP18, it is proposed that USP18 expression is suppressed in the late phase of ALI [45]. These findings suggest that GLN may have antiviral and anti-inflammatory capacities after ALI induction.

The compromised integrity of the alveolar–capillary barrier contributes to ALI. Neutrophil migration, excessive inflammation, oxidative stress, and cytotoxic mediators result in the loss of the integrity of the alveolar–capillary barrier [1]. The alveolar–capillary barrier is a thin membrane in the lungs composed of the capillary endothelium, a basement membrane, and the alveolar epithelium. The integrity of the capillary endothelium is maintained by the adherens junction protein VE-cadherin, which promotes cell adhesion and stabilizes the pulmonary endothelium. This stabilization is crucial for regulating vascular permeability and the extravasation of leukocytes [49]. Claudin-5, a tight junction protein, also plays a key role in controlling vascular permeability. Adherens and tight junctions are interconnected and share common features. Adherens junctions develop during the initial stages of intercellular contact and are essential for the formation of tight junctions [50]. In cases of acute lung injury, proinflammatory cytokines such as IL-6 and TNF-α stimulate the phosphorylation of VE-cadherin. This process leads to the disruption and misalignment of both VE-cadherin and Claudin-5, resulting in gaps between endothelial cells and a compromise of the barrier function [51]. In this experiment, the PG group exhibited lower plasma and BALF levels of IL-6 and TNF-α compared to the PS group. This phenomenon may help alleviate the disruption of VE-cadherin and Claudin-5 structures, thereby preserving the integrity of the capillary endothelium following poly(I:C)-induced lung injury. Claudin18.1 is a tight junction protein highly expressed by the alveolar epithelium. A previous study reported that mice lacking claudin18.1 exhibited a loss of epithelial integrity and lung injury [52]. Our results demonstrated that poly(I:C) stimulation downregulated the expressions of adherens/tight junction proteins on the capillary endothelium and alveolar epithelium, while GLN improved the integrity of the alveolar–capillary barrier in ALI.

Several mechanisms may be involved in the favorable effects of GLN administration after poly(I:C) stimulation. **First**, GLN reversed the depletion of plasma GLN levels, modulated a more balanced leukocyte distribution, and decreased leukocyte immigration. GLN is an essential substrate for rapidly proliferating immune cells. Previous studies found that cytokine secretion and the phagocytic capacity of neutrophils depend on GLN availability [53,54]. GLN promotes the antiviral function of M1 macrophages by boosting nitric oxide levels through increased arginine production [55]. Furthermore, GLN is able to protect neutrophils against apoptosis and inhibit migration to infection sites without inducing over-inflammation [14,56]. It is proposed that GLN provides more fuel sources to fulfill immune cell demands during viral infections. **Second**, GLN activated the TLR-3-signal pathway by upregulating type I and type III IFN-associated components that possessed an antiviral capacity and prevented excessive inflammation. A previous study found that GLN can inhibit NF-κB activation and downstream target gene expressions, thus preventing the occurrence of ARDS [57]. In addition, GLN is one of the ligands of peroxisome proliferator-activated receptor-γ which has anti-inflammatory properties [58]. Reduced inflammatory mediator levels in BALF showed that the inflammatory response had been attenuated in the lungs. **Third**, GLN administration improved the integrity of the alveolar–capillary barrier. An in vitro study found that GLN alleviated ROS-induced apoptosis in intestinal epithelial cells by regulating glutathione redox homeostasis [59]. A previous study showed that GLN supplementation attenuated ethanol-induced bronchoalveolar epithelial permeability and had a protective effect against alveolar epithelial barrier dysfunction [60]. GLN serves as the precursor for the endogenous antioxidant glutathione [61]. Since inflammation and oxidative stress can compromise pulmonary barrier function, a reduction in the production of inflammatory mediators may help maintain normal capillary barrier function. Histological evidence also indicated that lung injury was less severe after poly(I:C) stimulation when GLN was administered. In this study, various different markers involving inflammation, TLR-3 signaling, neutrophil infiltration, and barrier integrity were evaluated at three time points post-poly(I:C). Since the metabolic changes after poly(I:C) instillation for each specific marker differ, the influences of the GLN intervention may not be comparable among groups at different time points. However, there is a consistency that GLN administration reduces inflammation, activates the TLR-3 antiviral pathway, decreases neutrophil infiltration, and improves barrier integrity in the lungs at either one or all time points after poly(I:C) stimulation.

This study has two major limitations that should be addressed in future research. **First**, while the study used poly(I:C) to mimic the TLR-3 pathway activated by single-stranded RNA (ssRNA) viruses, real viral infections involve several critical stages: attachment, penetration, uncoating, replication, assembly, and egress. Each of these stages presents unique challenges for the immune system. Therefore, future research should focus on using actual viral infections to evaluate the effects of GLN. **Second**, in this experimental design, GLN was administered immediately one hour after the lung was insulted with poly(I:C). However, in a clinical setting, patients typically present post-infection and may have severe symptoms. We suggest that GLN should be given to patients as early as possible, but further studies are needed to demonstrate that GLN supplementation can also be effective even at the severe stage of lung infection.

## 5. Conclusions

**Figure 9:**
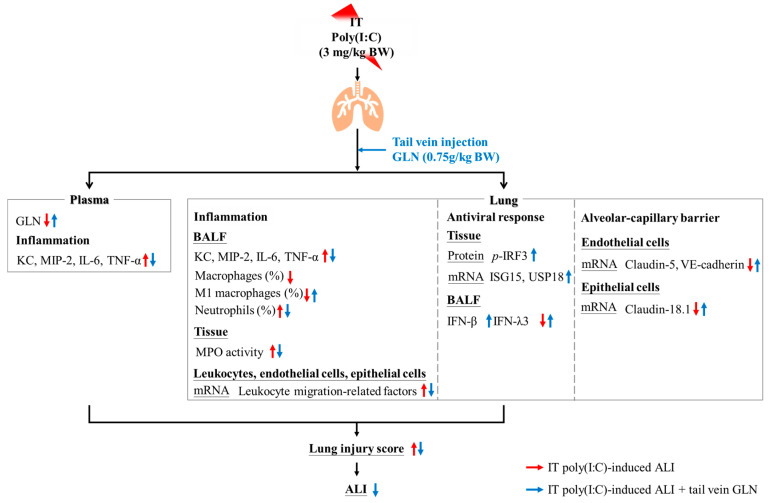
Illustration of the potential mechanisms of glutamine (GLN) administration after poly(I:C) stimulation. The red arrow indicates the effects of poly(I:C) stimulation, while the blue arrow illustrates the effects of GLN administration following poly(I:C) stimulation. ALI, acute lung injury; BW, body weight; GLN, glutamine; IFN-β, interferon-β; IFN-λ3, interferon-λ3; IL-6, interleukin-6; ISG15, interferon-stimulated gene 15; IT, intratracheal injection; KC, keratinocyte-derived chemokine; MIP-2, macrophage inflammatory protein-2; *p*-IRF3, phosphorylated interferon-regulatory factor 3; MPO, myeloperoxidase; TNF-α, tumor necrosis factor-α; USP18, ubiquitin specific peptidase 18; VE-cadherin, vascular endothelial cadherin.

In summary, the findings indicate that poly(I:C) stimulation led to inflammation, neutrophil infiltration, and impaired barrier integrity in the lungs. The IV administration of GLN after poly(I:C) stimulation ameliorated the inflammation and injury in the lung by activating the TLR-3 antiviral signaling pathway, inhibiting neutrophil infiltration, and enhancing alveolar–capillary barrier integrity. (The proposed mechanisms are presented in Figure 9). These findings provide foundational insights and suggest that a single dose of IV GLN administration after respiratory virus infection may have potential therapeutic significance in mitigating systemic inflammation and modulating ssRNA virus-related TLR-3 pathway-induced acute respiratory distress syndrome or other critical illnesses.

## Data Availability

The original contributions presented in this study are included in the article/Supplementary Materials. Further inquiries can be directed to the corresponding author.

## Supplementary Materials

The following supporting information can be downloaded at https://www.mdpi.com/article/10.3390/nu17101700/s1, Table S1. Sequences of oligonucleotide primers used for PCR amplification; Table S2. The body weights (BWs) and the weights of lung tissues; Figure S1. The illustration of the fluorescence minus one (FMO) control for gating the cell population.

## Abbreviations

The following abbreviations are used in this manuscript:

ALI	Acute lung injury
ARDS	Acute respiratory distress syndrome
BALF	Bronchoalveolar lavage fluid
BW	Body weight
CXCR2	C-X-C motif chemokine receptor 2
dsRNA	Double-stranded RNA
ELISA	Enzyme-linked immunosorbent assay
GLN	Glutamine
H&E	Hematoxylin and eosin
ICAM-1	Intercellular adhesion molecule 1
IFN	Interferon
IL	Interleukin
IRF3	IFN-regulatory factor 3
ISG15	IFN-stimulated gene 15
IV	intravenous
KC	Keratinocyte-derived chemokine
LPS	Lipopolysaccharide
MIP-2	Macrophage inflammatory protein-2
MPO	Myeloperoxidase
PBS	Phosphate-buffered saline
ROS	Reactive oxygen species
TLR	Toll-like receptor
TNF-α	Tumor necrosis factor-α
TRIF	Toll-interleukin-1 receptor-domain-containing adapter-inducing interferon-β
UPLC	Ultraperformance liquid chromatography
USP18	Ubiquitin-specific peptidase 18
VE-cadherin	Vascular endothelial-cadherin

## References

[1] Mowery N.T., Terzian W.T.H., Nelson A.C. (2020). Acute lung injury. Curr. Probl. Surg..

[2] Hodinka R.L. (2016). Respiratory RNA Viruses. Microbiol. Spectr..

[3] Hsieh P.C., Wu Y.K., Yang M.C., Su W.L., Kuo C.Y., Lan C.C. (2022). Deciphering the role of damage-associated molecular patterns and inflammatory responses in acute lung injury. Life Sci..

[4] Cheng P., Li S., Chen H. (2021). Macrophages in Lung Injury, Repair, and Fibrosis. Cells.

[5] Yamashita M., Niisato M., Kawasaki Y., Karaman S., Robciuc M.R., Shibata Y., Ishida Y., Nishio R., Masuda T., Sugai T. (2022). VEGF-C/VEGFR-3 signalling in macrophages ameliorates acute lung injury. Eur. Respir. J..

[6] Rosales C. (2018). Neutrophil: A Cell with Many Roles in Inflammation or Several Cell Types?. Front. Physiol..

[7] Yang S.C., Tsai Y.F., Pan Y.L., Hwang T.L. (2021). Understanding the role of neutrophils in acute respiratory distress syndrome. Biomed. J..

[8] Akgul C., Moulding D.A., Edwards S.W. (2001). Molecular control of neutrophil apoptosis. FEBS Lett..

[9] Chen Y., Lin J., Zhao Y., Ma X., Yi H. (2021). Toll-like receptor 3 (TLR3) regulation mechanisms and roles in antiviral innate immune responses. J. Zhejiang Univ. Sci. B.

[10] Gibbert K., Francois S., Sigmund A.M., Harper M.S., Barrett B.S., Kirchning C.J., Lu M., Santiago M.L., Dittmer U. (2014). Friend retrovirus drives cytotoxic effectors through Toll-like receptor 3. Retrovirology.

[11] Mohajeri M., Horriatkhah E., Mohajery R. (2021). The effect of glutamine supplementation on serum levels of some inflammatory factors, oxidative stress, and appetite in COVID-19 patients: A case-control study. Inflammopharmacology.

[12] Bharadwaj S., Singh M., Kirtipal N., Kang S.G. (2020). SARS-CoV-2 and Glutamine: SARS-CoV-2 Triggered Pathogenesis via Metabolic Reprograming of Glutamine in Host Cells. Front. Mol. Biosci..

[13] Kim H. (2011). Glutamine as an immunonutrient. Yonsei Med. J..

[14] Santos A.C.A., Hebeba C.B., Hastreiter A.A., de Oliveira D.C., Naoto Makiyama E., Farsky S.H.P., Borelli P., Fock R.A. (2019). Exogenous glutamine impairs neutrophils migration into infections sites elicited by lipopolysaccharide by a multistep mechanism. Amino Acids.

[15] Hou Y.C., Chiu W.C., Yeh C.L., Yeh S.L. (2012). Glutamine modulates lipopolysaccharide-induced activation of NF-κB via the Akt/mTOR pathway in lung epithelial cells. Am. J. Physiol. Lung Cell Mol. Physiol..

[16] Yeh C.L., Hsu C.S., Yeh S.L., Chen W.J. (2005). Dietary glutamine supplementation modulates Th1/Th2 cytokine and interleukin-6 expressions in septic mice. Cytokine.

[17] Yeh C.L., Su L.H., Wu J.M., Yang P.J., Lee P.C., Chen P.D., Huang C.C., Hsieh D.Y., Wang H.J., Yeh S.L. (2020). Effects of the Glutamine Administration on T Helper Cell Regulation and Inflammatory Response in Obese Mice Complicated with Polymicrobial Sepsis. Mediat. Inflamm..

[18] Hu Y.M., Yeh C.L., Pai M.H., Lee W.Y., Yeh S.L. (2014). Glutamine administration modulates lung γδ T lymphocyte expression in mice with polymicrobial sepsis. Shock.

[19] Amemiya K., Dankmeyer J.L., Bernhards R.C., Fetterer D.P., Waag D.M., Worsham P.L., DeShazer D. (2021). Activation of Toll-Like Receptors by Live Gram-Negative Bacterial Pathogens Reveals Mitigation of TLR4 Responses and Activation of TLR5 by Flagella. Front. Cell Infect. Microbiol..

[20] Stowell N.C., Seideman J., Raymond H.A., Smalley K.A., Lamb R.J., Egenolf D.D., Bugelski P.J., Murray L.A., Marsters P.A., Bunting R.A. (2009). Long-term activation of TLR3 by poly(I:C) induces inflammation and impairs lung function in mice. Respir. Res..

[21] Ulbrich H., Eriksson E.E., Lindbom L. (2003). Leukocyte and endothelial cell adhesion molecules as targets for therapeutic interventions in inflammatory disease. Trends Pharmacol. Sci..

[22] Nolte D., Kuebler W.M., Muller W.A., Wolff K.D., Messmer K. (2004). Attenuation of leukocyte sequestration by selective blockade of PECAM-1 or VCAM-1 in murine endotoxemia. Eur. Surg. Res..

[23] Yeh C.L., Wu J.M., Chen K.Y., Wu M.H., Yang P.J., Lee P.C., Chen P.D., Kuo T.C., Yeh S.L., Lin M.T. (2024). Calcitriol attenuates poly(I:C)-induced lung injury in obese mice via modulating toll-like receptor 3- and renin-angiotensin system-associated signal pathways. Int. Immunopharmacol..

[24] Oliveira G.P., Oliveira M.B., Santos R.S., Lima L.D., Dias C.M., Ab’Saber A.M., Teodoro W.R., Capelozzi V.L., Gomes R.N., Bozza P.T. (2009). Intravenous glutamine decreases lung and distal organ injury in an experimental model of abdominal sepsis. Crit. Care.

[25] Huang C., Wang Y., Li X., Ren L., Zhao J., Hu Y., Zhang L., Fan G., Xu J., Gu X. (2020). Clinical features of patients infected with 2019 novel coronavirus in Wuhan, China. Lancet.

[26] Dawood F.S., Iuliano A.D., Reed C., Meltzer M.I., Shay D.K., Cheng P.Y., Bandaranayake D., Breiman R.F., Brooks W.A., Buchy P. (2012). Estimated global mortality associated with the first 12 months of 2009 pandemic influenza A H1N1 virus circulation: A modelling study. Lancet Infect. Dis..

[27] Curi R., Newsholme P., Pithon-Curi T.C., Pires-de-Melo M., Garcia C., Homem-de-Bittencourt Júnior P.I., Guimarães A.R. (1999). Metabolic fate of glutamine in lymphocytes, macrophages and neutrophils. Braz. J. Med. Biol. Res..

[28] Robinson N., Ganesan R., Hegedűs C., Kovács K., Kufer T.A., Virág L. (2019). Programmed necrotic cell death of macrophages: Focus on pyroptosis, necroptosis, and parthanatos. Redox Biol..

[29] Li K., McCaw J.M., Cao P. (2021). Modelling within-host macrophage dynamics in influenza virus infection. J. Theor. Biol..

[30] Yu S., Ge H., Li S., Qiu H.J. (2022). Modulation of Macrophage Polarization by Viruses: Turning Off/On Host Antiviral Responses. Front. Microbiol..

[31] Cassetta L., Kajaste-Rudnitski A., Coradin T., Saba E., Della Chiara G., Barbagallo M., Graziano F., Alfano M., Cassol E., Vicenzi E. (2013). M1 polarization of human monocyte-derived macrophages restricts pre and postintegration steps of HIV-1 replication. Aids.

[32] Puneet P., Moochhala S., Bhatia M. (2005). Chemokines in acute respiratory distress syndrome. Am. J. Physiol. Lung Cell Mol. Physiol..

[33] Griffith J.W., Sokol C.L., Luster A.D. (2014). Chemokines and chemokine receptors: Positioning cells for host defense and immunity. Annu. Rev. Immunol..

[34] Girbl T., Lenn T., Perez L., Rolas L., Barkaway A., Thiriot A., Del Fresno C., Lynam E., Hub E., Thelen M. (2018). Distinct Compartmentalization of the Chemokines CXCL1 and CXCL2 and the Atypical Receptor ACKR1 Determine Discrete Stages of Neutrophil Diapedesis. Immunity.

[35] Bednarczyk M., Stege H., Grabbe S., Bros M. (2020). β2 Integrins-Multi-Functional Leukocyte Receptors in Health and Disease. Int. J. Mol. Sci..

[36] Reutershan J., Morris M.A., Burcin T.L., Smith D.F., Chang D., Saprito M.S., Ley K. (2006). Critical role of endothelial CXCR2 in LPS-induced neutrophil migration into the lung. J. Clin. Investig..

[37] Lin W.C., Fessler M.B. (2021). Regulatory mechanisms of neutrophil migration from the circulation to the airspace. Cell Mol. Life Sci..

[38] Steichen A.L., Simonson T.J., Salmon S.L., Metzger D.W., Mishra B.B., Sharma J. (2015). Alarmin function of galectin-9 in murine respiratory tularemia. PLoS ONE.

[39] Effah C.Y., Drokow E.K., Agboyibor C., Ding L., He S., Liu S., Akorli S.Y., Nuamah E., Sun T., Zhou X. (2021). Neutrophil-Dependent Immunity During Pulmonary Infections and Inflammations. Front. Immunol..

[40] Mullane K.M., Kraemer R., Smith B. (1985). Myeloperoxidase activity as a quantitative assessment of neutrophil infiltration into ischemic myocardium. J. Pharmacol. Methods.

[41] Park A., Iwasaki A. (2020). Type I and Type III Interferons—Induction, Signaling, Evasion, and Application to Combat COVID-19. Cell Host Microbe.

[42] Han L., Zhuang M.W., Deng J., Zheng Y., Zhang J., Nan M.L., Zhang X.J., Gao C., Wang P.H. (2021). SARS-CoV-2 ORF9b antagonizes type I and III interferons by targeting multiple components of the RIG-I/MDA-5-MAVS, TLR3-TRIF, and cGAS-STING signaling pathways. J. Med. Virol..

[43] Kessler D.S., Levy D.E., Darnell J.E. (1988). Two interferon-induced nuclear factors bind a single promoter element in interferon-stimulated genes. Proc. Natl. Acad. Sci. USA.

[44] Jiménez Fernández D., Hess S., Knobeloch K.P. (2019). Strategies to Target ISG15 and USP18 Toward Therapeutic Applications. Front. Chem..

[45] Zhang X., Bogunovic D., Payelle-Brogard B., Francois-Newton V., Speer S.D., Yuan C., Volpi S., Li Z., Sanal O., Mansouri D. (2015). Human intracellular ISG15 prevents interferon-α/β over-amplification and auto-inflammation. Nature.

[46] Jewell N.A., Cline T., Mertz S.E., Smirnov S.V., Flaño E., Schindler C., Grieves J.L., Durbin R.K., Kotenko S.V., Durbin J.E. (2010). Lambda interferon is the predominant interferon induced by influenza A virus infection in vivo. J. Virol..

[47] Dellgren C., Gad H.H., Hamming O.J., Melchjorsen J., Hartmann R. (2009). Human interferon-lambda3 is a potent member of the type III interferon family. Genes. Immun..

[48] Bolen C.R., Ding S., Robek M.D., Kleinstein S.H. (2014). Dynamic expression profiling of type I and type III interferon-stimulated hepatocytes reveals a stable hierarchy of gene expression. Hepatology.

[49] Vestweber D. (2008). VE-cadherin: The major endothelial adhesion molecule controlling cellular junctions and blood vessel formation. Arterioscler. Thromb. Vasc. Biol..

[50] Dejana E., Tournier-Lasserve E., Weinstein B.M. (2009). The control of vascular integrity by endothelial cell junctions: Molecular basis and pathological implications. Dev. Cell.

[51] Grommes J., Soehnlein O. (2011). Contribution of neutrophils to acute lung injury. Mol. Med..

[52] Kotton D.N. (2018). Claudin-18: Unexpected regulator of lung alveolar epithelial cell proliferation. J. Clin. Investig..

[53] Yaqoob P., Calder P.C. (1998). Cytokine production by human peripheral blood mononuclear cells: Differential senstivity to glutamine availability. Cytokine.

[54] Field C.J., Johnson I.R., Schley P.D. (2002). Nutrients and their role in host resistance to infection. J. Leukoc. Biol..

[55] Murphy C., Newsholme P. (1998). Importance of glutamine metabolism in murine macrophages and human monocytes to L-arginine biosynthesis and rates of nitrite or urea production. Clin. Sci..

[56] Pithon-Curi T.C., Schumacher R.I., Freitas J.J., Lagranha C., Newsholme P., Palanch A.C., Doi S.Q., Curi R. (2003). Glutamine delays spontaneous apoptosis in neutrophils. Am. J. Physiol. Cell Physiol..

[57] Singleton K.D., Beckey V.E., Wischmeyer P.E. (2005). Glutamine prevents activation of nf-kappab and stress kinase pathways, attenuates inflammatory cytokine release, and prevents acute respiratory distress syndrome (ARDS) following sepsis. Shock.

[58] Sato N., Moore F.A., Kone B.C., Zou L., Smith M.A., Childs M.A., Moore-Olufemi S., Schultz S.G., Kozar R.A. (2006). Differential induction of PPAR-gamma by luminal glutamine and iNOS by luminal arginine in the rodent postischemic small bowel. Am. J. Physiol. Gastrointest. Liver Physiol..

[59] Liu N., Ma X., Luo X., Zhang Y., He Y., Dai Z., Yang Y., Wu G., Wu Z. (2018). l-Glutamine Attenuates Apoptosis in Porcine Enterocytes by Regulating Glutathione-Related Redox Homeostasis. J. Nutr..

[60] Zhang Y.L., Li Q.Q., Guo W., Huang Y., Yang J. (2007). Effects of chronic ethanol ingestion on tight junction proteins and barrier function of alveolar epithelium in the rat. Shock.

[61] Roth E., Oehler R., Manhart N., Exner R., Wessner B., Strasser E., Spittler A. (2002). Regulative potential of glutamine--relation to glutathione metabolism. Nutrition.

