# Supplementary material for: Glutamine Administration Attenuates Poly(I:C)-Induced Lung Injury by Reducing Neutrophil Infiltration and Activating the TLR-3 Antiviral Pathway
Source: Nutrients. 2025 May 16;17(10):1700. doi: 10.3390/nu17101700 (PMC12113983; doi:10.3390/nu17101700)
Supplement: Supplementary file 1 [file nutrients-17-01700-s001.zip › nutrients-3579356-supplementary.pdf]

Table S1. Sequences of oligonucleotide primers used for PCR amplification

Gene name	Primer sequence (5’→3’)	Accession no.
**β2-integrin**	**F:** TTCCTGGTGCCAGAAGCTGAA<br>**R:** AAAGGCAGCACCGTCTTGTC	NM_008404.5
**CD47**	**F:** TTGTCGCTTCCAACCAGAGG<br>**R:** CTTCCAGCTGTGAGTCGTGA	NM_001368415.1
**Claudin-5**	**F:** GCCTTCCTGGACCACAACAT<br>**R:** CGCCGGTCAAGGTAACAAAG	NM_013805.4
**Claudin-18.1**	**F:** CCTGACACCAGATGACAGCA<br>**R:** TTCCTCCTGCACACAGGTTG	NM_019815.3
**CXCR2**	**F:** GAGCCACTCTGCTCACAAAC<br>**R:** GCAGAGTCACCAGGACGTAT	NM_009909.3
**Galectin-9**	**F:** TCAAGGTGATGGTGAACAAGAAA<br>**R:** GATGGTGTCCACGAGGTGGTA	NM_010708.2
**ICAM-1**	**F:** TGTCAGCCACCATGCCTTAG<br>**R:** CAGCTTGCACGACCCTTCTA	NM_010493.3
**ISG15**	**F:** TGGTACAGAACTGCAGCGAG<br>**R:** AGCCAGAACTGGTCTTCGTG	NM_015783.3
**USP18**	**F:** GATCACGGACACAGACTTGACAG<br>**R:** ACTCTTTGGGCTGGACGAAAC	NM_011909.2
**VE-cadherin**	**F:** CCTGAGGCAATCAACTGTGC<br>**R:** GAGGAGCTGATCTTGTCCGT	NM_009868.4
**GAPDH**	**F:** AACGACCCCTTCATTGAC<br>**R:** TCCACGACATACTCAGCAC	M32599.1

CD47, cluster of differentiation 47; CXCR2, C-X-C Motif chemokine receptor 2; ICAM-1, intercellular adhesion molecule 1; ISG15, interferon-stimulated gene 15; USP18, ubiquitin specific

peptidase 18; VE-cadherin, vascular endothelial cadherin; GAPDH, glyceraldehyde 3-phosphate dehydrogenase.

Table S2. The body weights (BWs) and the weights of lung tissues.

	NC	SH	PS4h	PG4h	PS12h	PG12h	PS24h	PG24h
BWs (g)	25.74 ± 0.44	26.13 ± 0.42	26.34 ± 0.51	26.21 ± 0.51	26.02 ± 0.39	26.13 ± 0.47	25.8 ± 0.45	26.01 ± 0.43
Lung/BW	0.0111 ± 0.0002	0.0112 ± 0.0001	0.0117 ± 0.0004	0.0112 ± 0.0003	0.0128 ± 0.0005*	0.0119 ± 0.0002	0.0138 ± 0.0006*	0.0126 ± 0.0002

NC group, normal control group; SH group, intratracheally administered with saline; PS group, intratracheally administered with poly(I:C) and an IV injection with saline; PG group, intratracheally administered with poly(I:C) and an IV injection with glutamine. The data are represented as the mean ± standard error of the mean (SEM). Student's *t*-test was used to analyze differences between the NC and SH group. Two-way analysis of variance (ANOVA) followed by Bonferroni *post-hoc* test was used to analyze differences among the SH and PS group at three time points, and between the PS and PG groups at the same time points. * Significantly different from the SH group ($p < 0.05$).

(A).

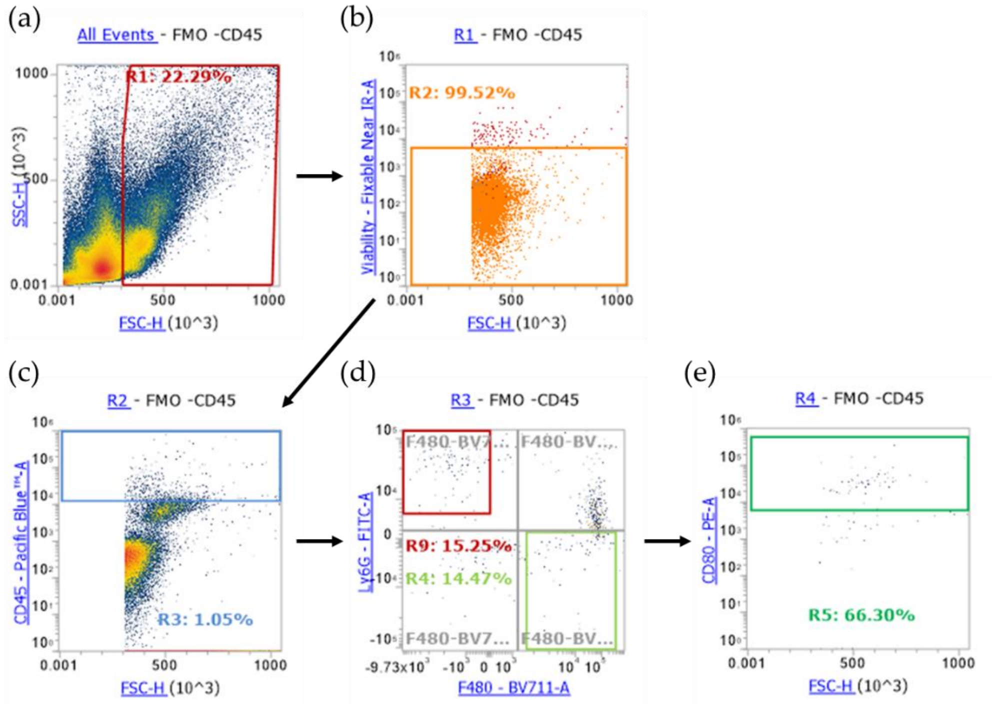
(a)
All Events - FMO -CD45
R1: 22.29%
SSC-H (10^3)
FSC-H (10^3)
(b)
R1 - FMO -CD45
R2: 99.52%
Viability - Fixable Near IR-A
FSC-H (10^3)
(c)
R2 - FMO -CD45
CD45 - Pacific Blue™-A
R3: 1.05%
FSC-H (10^3)
(d)
R3 - FMO -CD45
R9: 15.25%
R4: 14.47%
Ly6G - FITC-A
F480 - BV711-A
(e)
R4 - FMO -CD45
R5: 66.30%
CD80 - PE-A
FSC-H (10^3)

(B).

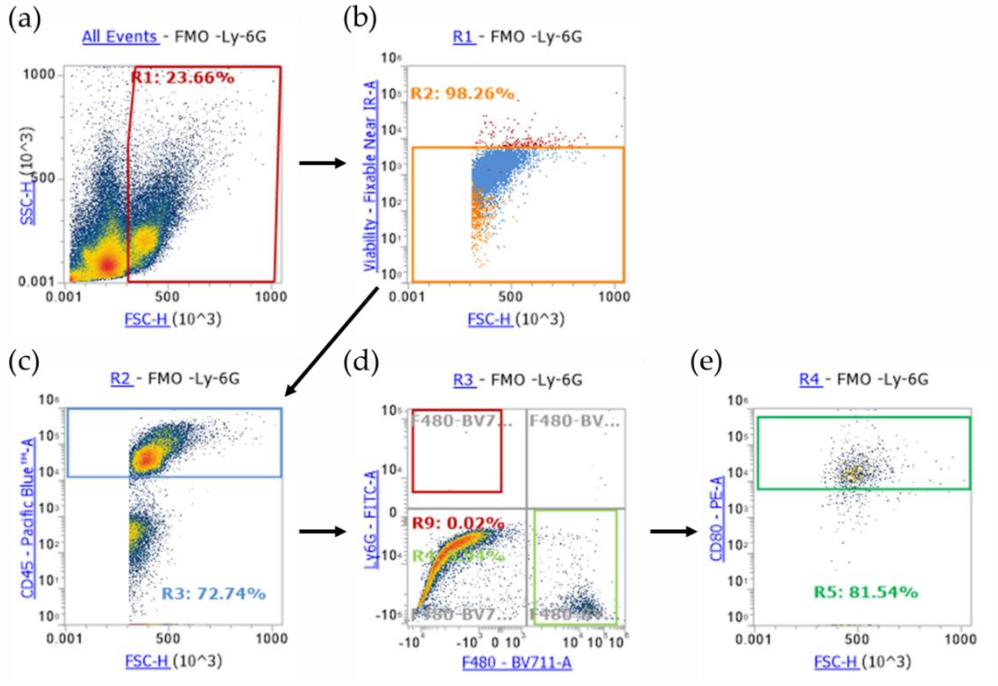
(a)
All Events - FMO -Ly-6G
R1: 23.66%
SSC-H (10^3)
FSC-H (10^3)
(b)
R1 - FMO -Ly-6G
R2: 98.26%
Viability - Fixable Near IR-A
FSC-H (10^3)
(c)
R2 - FMO -Ly-6G
CD45 - Pacific Blue™-A
R3: 72.74%
FSC-H (10^3)
(d)
R3 - FMO -Ly-6G
R9: 0.02%
Ly6G - FITC-A
F480 - BV711-A
(e)
R4 - FMO -Ly-6G
R5: 81.54%
CD80 - PE-A
FSC-H (10^3)

(C).

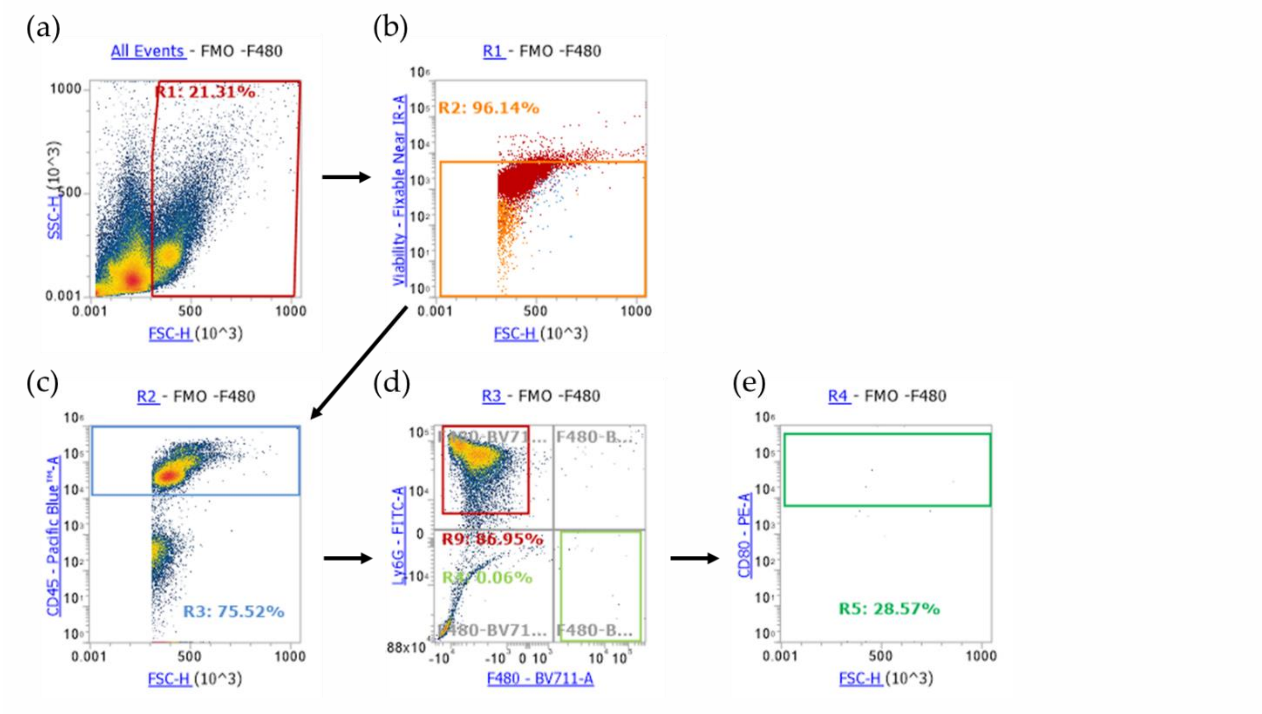
(a)
All Events - FMO -F480
R1: 21.31%
SSC-H (10^3)
FSC-H (10^3)
(b)
R1 - FMO -F480
R2: 96.14%
Viability - Fixable Near IR-A
FSC-H (10^3)
(c)
R2 - FMO -F480
CD45 - Pacific Blue™-A
R3: 75.52%
FSC-H (10^3)
(d)
R3 - FMO -F480
R9: 86.95%
Ly6G - FITC-A
F480 - BV711-A
(e)
R4 - FMO -F480
CD80 - PE-A
R5: 28.57%
FSC-H (10^3)

(D).

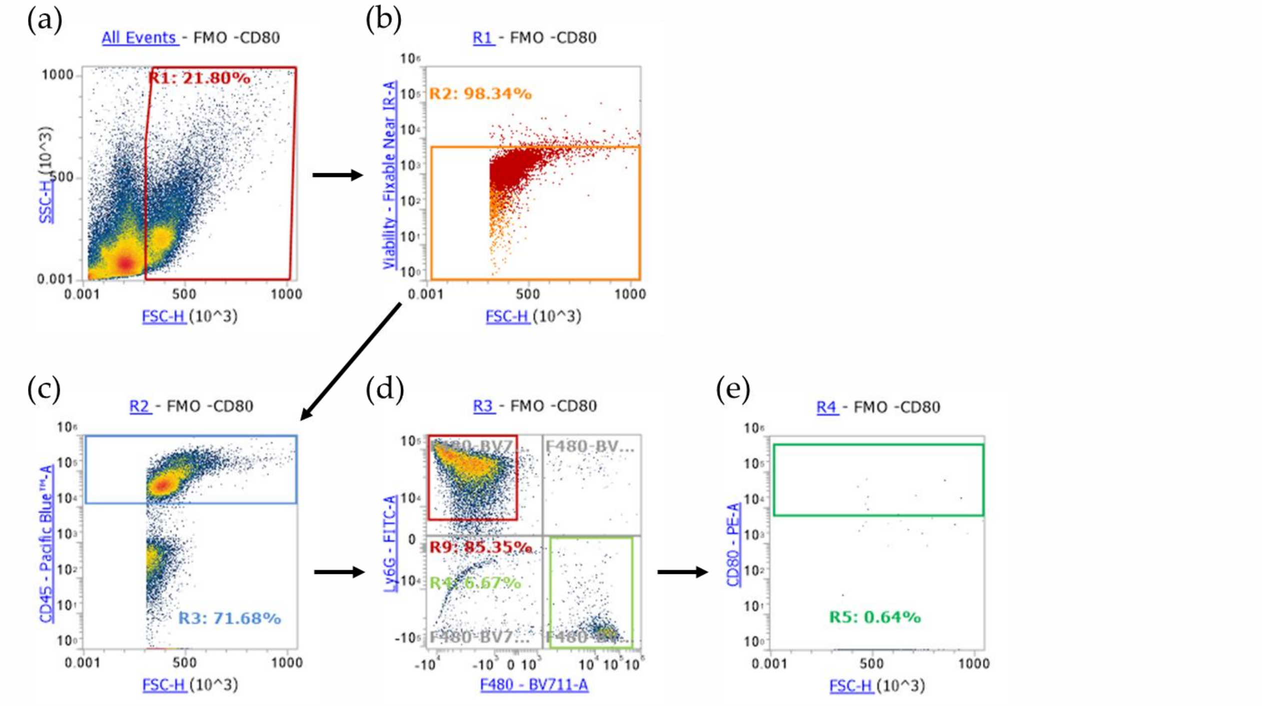
(a)
All Events - FMO -CD80
R1: 21.80%
SSC-H (10^3)
FSC-H (10^3)
(b)
R1 - FMO -CD80
R2: 98.34%
Viability - Fixable Near IR-A
FSC-H (10^3)
(c)
R2 - FMO -CD80
CD45 - Pacific Blue™-A
R3: 71.68%
FSC-H (10^3)
(d)
R3 - FMO -CD80
R9: 85.35%
Ly6G - FITC-A
F480 - BV711-A
(e)
R4 - FMO -CD80
CD80 - PE-A
R5: 0.64%
FSC-H (10^3)

(E).

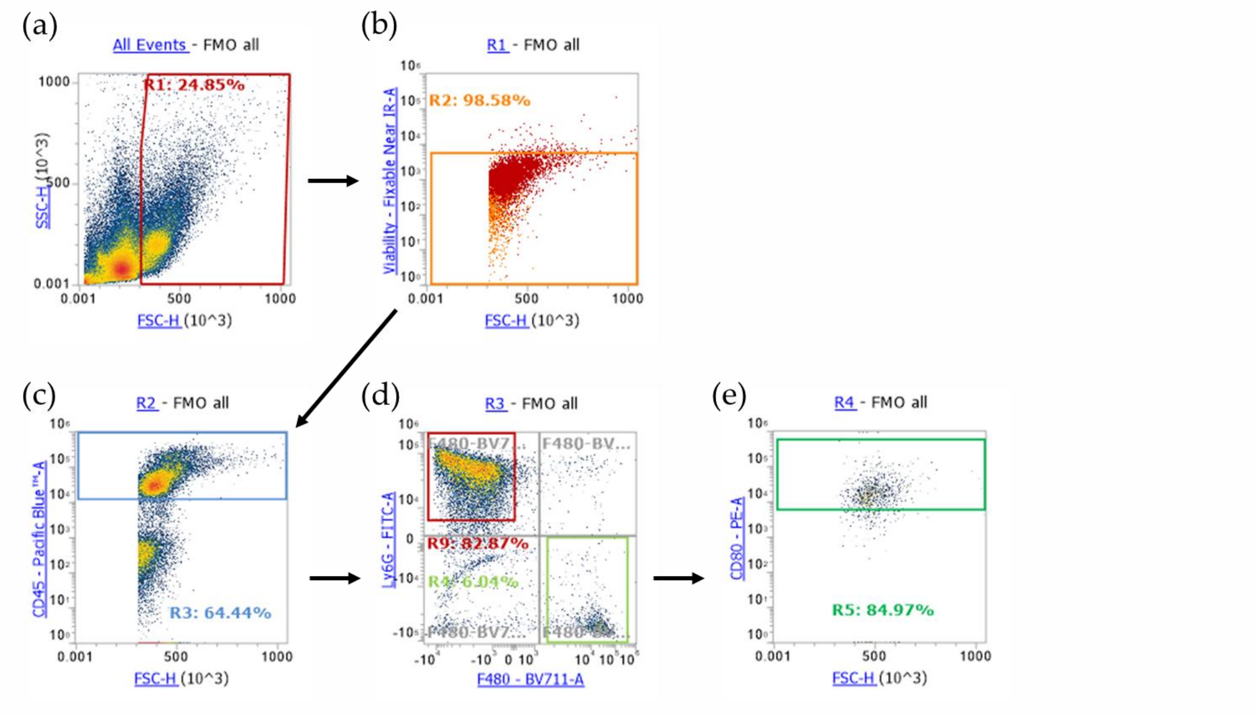

Figure S1. The illustration of fluorescence minus one (FMO) control for gating cell population. FMO control was conducted by removing one antibody once a time. In this study, we eliminated (A) CD45.2-Pacific blue (PB), (B) Ly-6G-fluorescein (FITC), (C) F4/80-brilliant violet 711 (BV711) and (D) CD80-phycoerythrin (PE), respectively. The representative gating strategy was demonstrated in (D). The R1 region indicated that the cells were gated to eliminate debris. Next, live cells were selected using a cell viability dye from the R1 region, as shown in the orange square in panel (b). In panel (c), the antibody CD45 was utilized to identify leukocytes among the live cells, with the blue square highlighting $CD45^+$ cells. Subsequently, Ly6G and F4/80 antibodies were applied to gate neutrophils ($CD45^+$/$Ly6G^+$) and macrophages ($CD45^+$/$F4/80^+$) in panel (d), respectively. Finally, panel (e) displays the $CD45.2^+$/$F4/80^+$/$CD80^+$ cells that were selected from the $CD45^+$/$F4/80^+$ population identified in panel (d).
